# Transformation of pancreatic nonfunctioning neuroendocrine tumor into metastatic insulinoma: A rare case report

**DOI:** 10.1002/ccr3.8152

**Published:** 2023-11-06

**Authors:** Venkata S. Buddhavarapu, Gagandeep Dhillon, Harpreet Singh Grewal, Brian Soles, Luke Halbur, Salim Surani, Rahul Kashyap

**Affiliations:** ^1^ Hospital Medicine, Banner Medical Group, Banner Health Mesa Arizona USA; ^2^ Department of Hospital Medicine University of Maryland Baltimore Washington Medical Center Glen Burnie Maryland USA; ^3^ Department of Radiology Florida State University College of Medicine Pensacola Florida USA; ^4^ Department of Pathology Pathology Specialists of Arizona Mesa Arizona USA; ^5^ Medical Oncology/Hematology Ironwood Cancer and Research Centers Mesa Arizona USA; ^6^ Department of Pharmacology & Medicine Texas A&M University College Station Texas USA; ^7^ Department of Research WellSpan Health York Pennsylvania USA

**Keywords:** case report, metastatic insulinoma, neoplasms, pancreatic neoplasms

## Abstract

Pancreatic neuroendocrine tumors can be classified as functional or nonfunctional based on hormone secretion. Management of each entity is different, with nonfunctional tumors being treated with traditional chemotherapy while functional tumors respond well to antihormonal therapy and immunologic agents. The conversion of one nonfunctional tumor into a functional tumor is an exceedingly rare event that complicates the overall management of these patients. In this report, we present the case of a 73‐year‐old woman who developed the conversion from a nonfunctional into a functional tumor and discuss the management options considered.

## Graphical abstract

Neuroendocrine tumor with Liver Metastasis with positive staining for chromogranin (A) and synaptophysin (B).

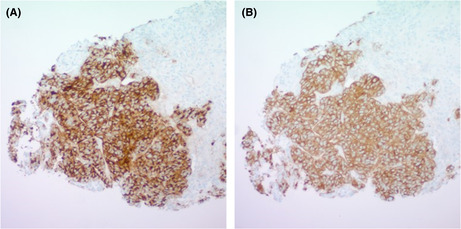

## 1 INTRODUCTION

Pancreatic neuroendocrine tumors (NETs) are a subset of NETs that mainly originate from the neuroendocrine system. They account for less than 10% of total NETs and have a general incidence of 0.5 per 100,000 persons per year.^1^ Only 10%–30% of these tumors are hormone‐secreting, with the majority being insulinomas. The tumors are often found incidentally in the pancreas or gastrointestinal (GI) system on imaging. Pathological specimens are generally positive for chromogranin A and synaptophysin but can additionally be positive for other serum markers such as Insulin, Gastrin, and Vasoactive Intestinal Peptide (VIP), depending on the subtype of NET.^2^ General management includes surgery for localized disease and surgery/chemotherapy in combination for extensive disease. Prognosis is poor for those with liver metastases, with an overall mortality rate >80%. Functional tumors are generally localized and rarely present as metastatic disease.^3^

Among functional NETs, insulinomas are the most common. Incidence is around 1–3 cases per million and is usually present in the pancreas over 99% of the time.^4^ Diagnosis is established with symptomatic hypoglycemia in the setting of elevated insulin and c‐peptide levels after a 72‐h fasting test. Blood glucose levels usually improve with glucagon or dextrose administration. These lesions are amenable to treatments such as alcohol ablation, radiofrequency ablation, embolization, or surgical resection. Surgical resection is the preferred option for isolated lesions as it is often curative.^5^ Symptomatic management of hypoglycemia involves dextrose administration and diazoxide. Diazoxide inhibits insulin release from insulinoma cells and is often the only effective measure prior to surgery.^6^ Somatostatin analogs such as octreotide, pasireotide, and lanreotide are also used in combination with diazoxide in more severe cases but can lead to episodes of hypoglycemia.^7^ Cases involving surgical management have a good prognosis.^6^, ^7^

Malignant insulinomas only account for about 5% of all insulinomas at presentation.^8^, ^9^ In patients with distant metastases, surgical resection with lymph node dissection improves prognosis. However, the overall difference is minimal, and the data are limited in these cases.^10^ Overall, these patients do quite poorly due to limited treatment options. There are very few case reports that discuss the conversion of nonfunctional metastatic NET into a malignant insulinoma,^11^ and these reports do suggest that patients have significant morbidity after this conversion. This case report presents such a conversion in a 73‐year‐old female patient.

## 2 CASE PRESENTATION

**FIGURE 1:**
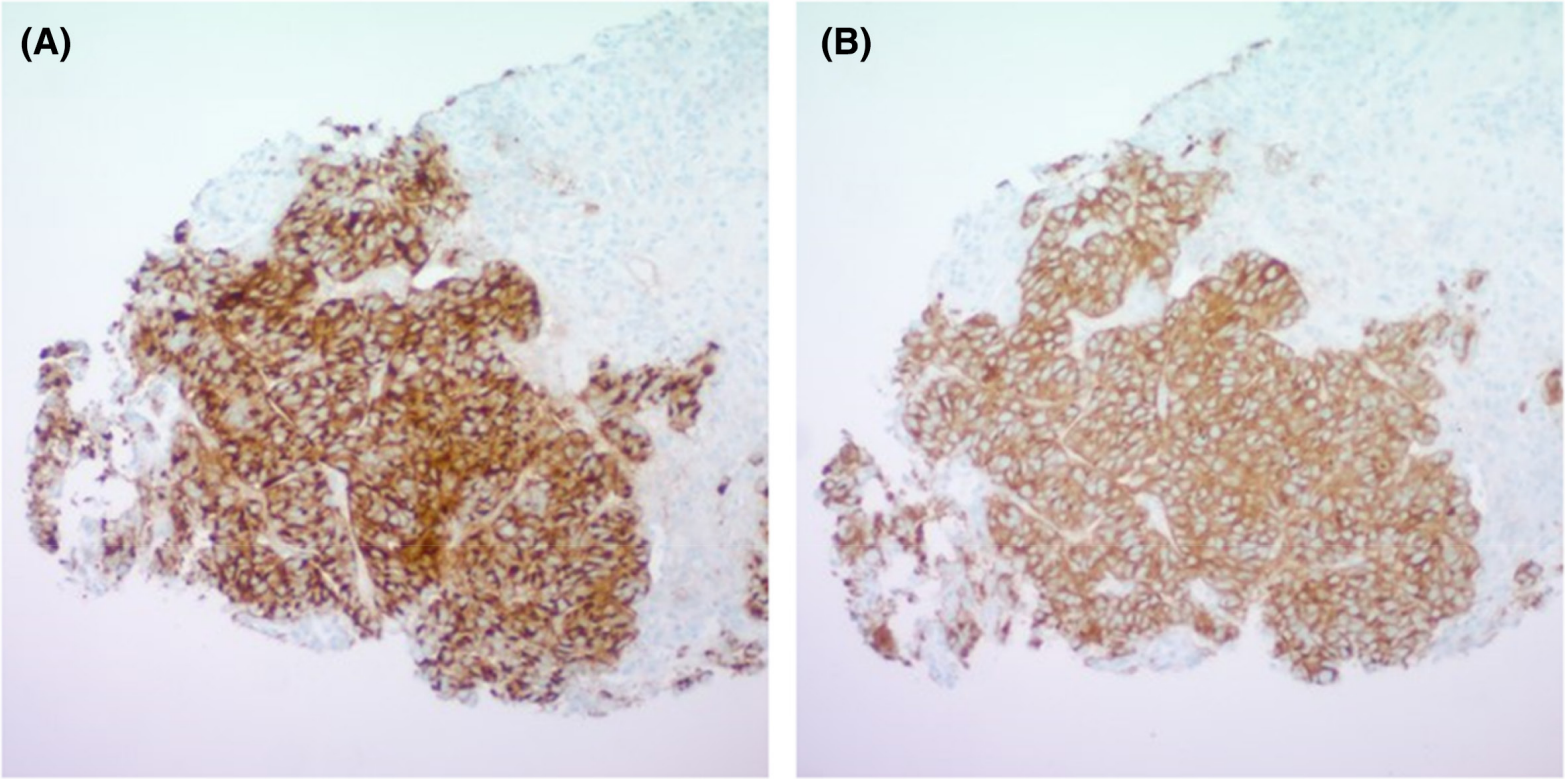
Liver tissue specimen showing positive staining for chromogranin (A) and synaptophysin (B).

Our case involves a 73‐year‐old woman with a prior medical history of type 2 diabetes mellitus and a recent diagnosis of nonhormone‐secreting high‐grade pancreatic NET with metastases to the liver, who presented for admission for bilateral pulmonary embolism. The pancreatic NET had been diagnosed from liver biopsy specimens showing positive stains for synaptophysin and chromogranin. The pathology images are shown in Figure 1A,B. The patient had been diagnosed 1 month prior to presentation and was undergoing further staging workup as an outpatient. She had not received any treatment prior to presentation.

**FIGURE 2:**
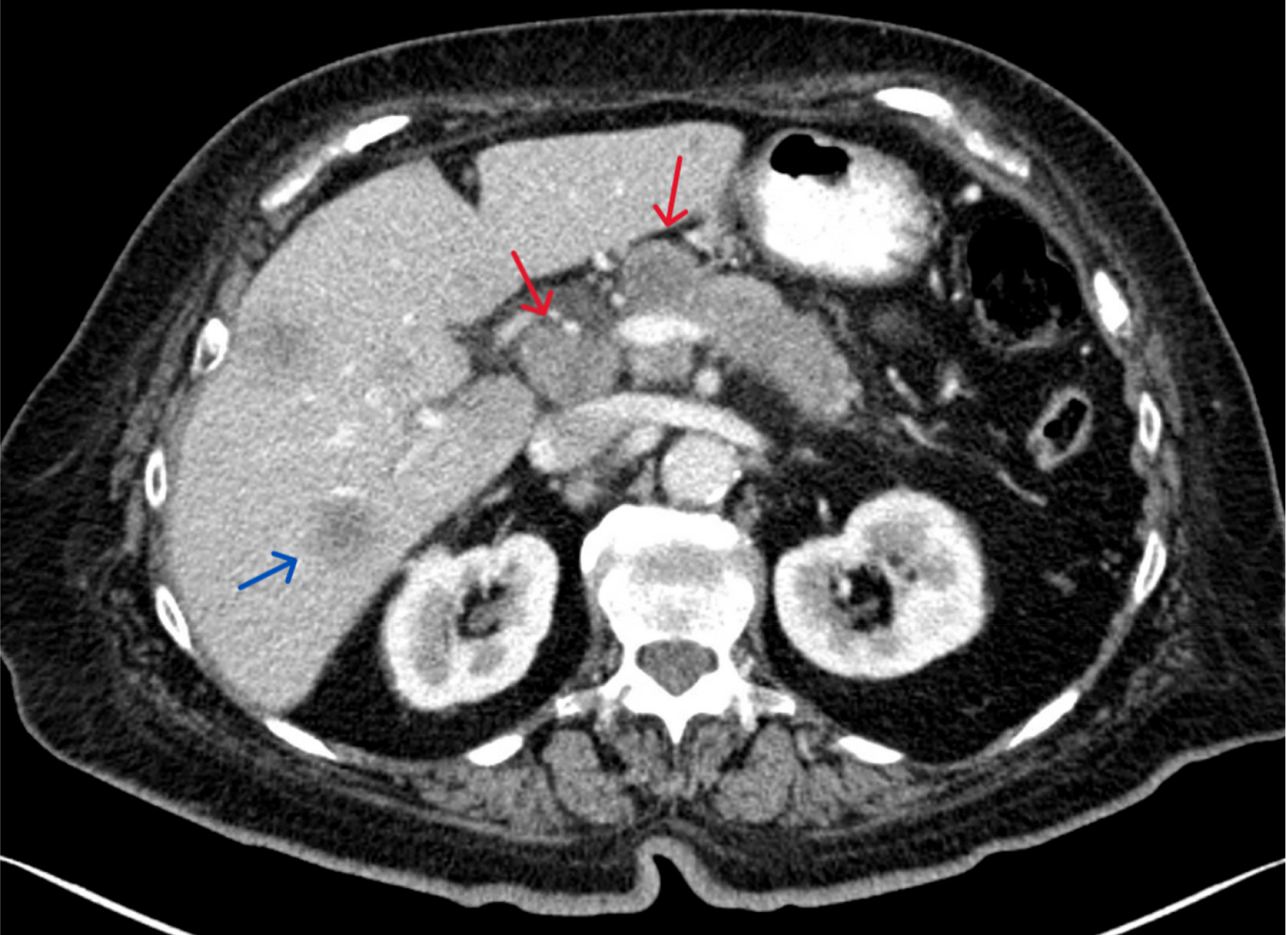
Axial post‐contrast CT image demonstrates the heterogeneously enhancing mass in the pancreatic head and proximal body (red arrows), compatible with the known primary neuroendocrine tumor. Incidentally, hepatic metastases are also partially seen (blue arrow).

**FIGURE 3:**
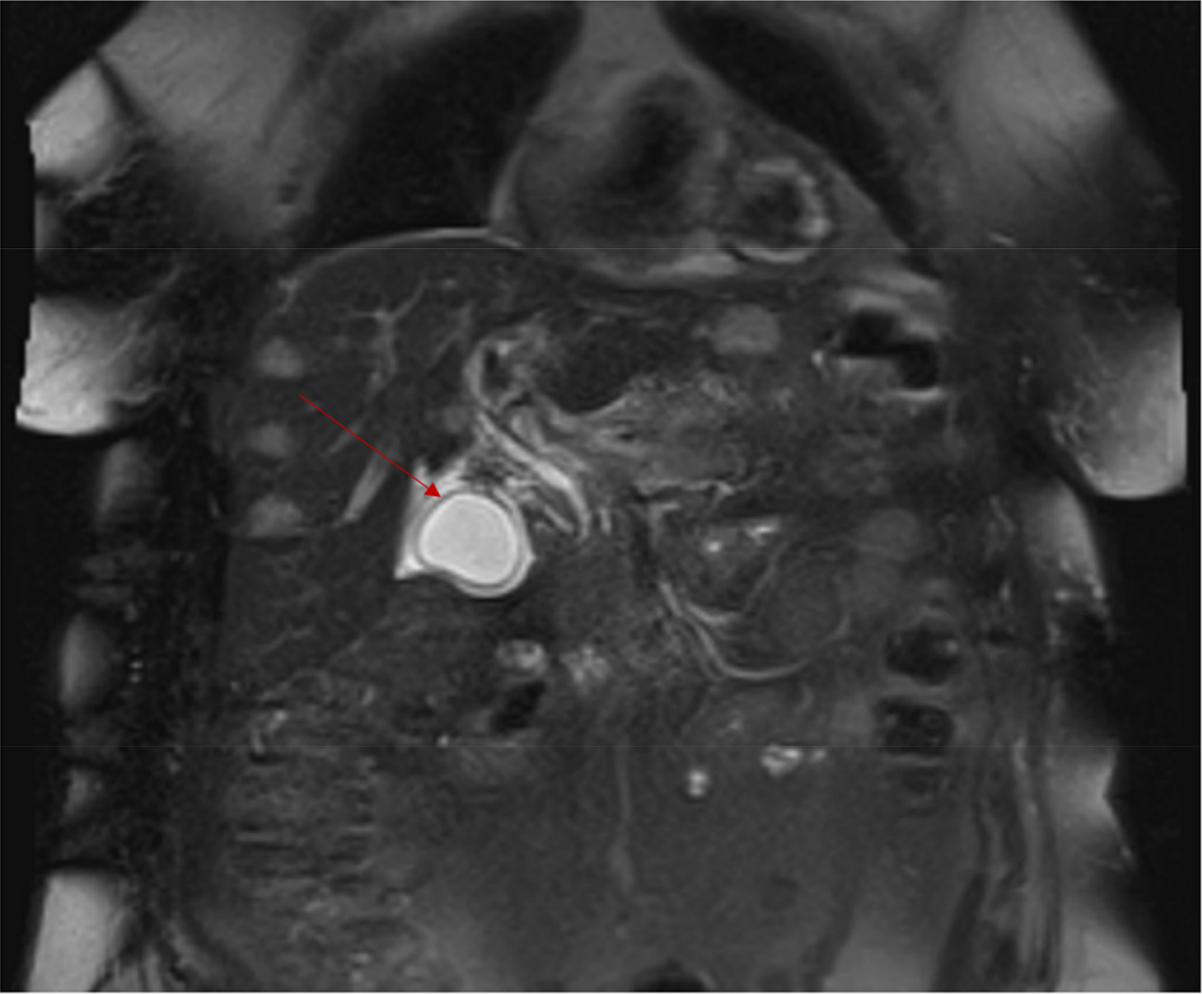
Coronal T2‐weighted image of the abdomen demonstrates a cystic lesion (red arrow) in the pancreatic head, corresponding to the patient's known pancreatic neuroendocrine tumor. Also seen are faintly T2WI hyperintense right hepatic lobe lesions, in keeping with the widely metastatic disease.

The patient's only complaints at presentation were generalized weakness, and she denied any respiratory complaints upon presentation. The patient previously had diabetes as well and was on a home regimen of Tresiba insulin 20 units daily and metformin 1000 mg twice daily. She had taken both medications the day before presentation, and neither was restarted on admission as the patient's blood glucose was near normal. Physical examination findings were unremarkable, including a normal oxygen saturation on room air. Laboratory findings revealed significant hypoglycemia with a blood glucose of 48 mg/dL. The patient was started on IV dextrose 5% with normal saline at 125 mL/h and IV heparin infusion at 18 units/kg/h and was subsequently admitted for further management. Of note, the patient's pancreatic tumor prior to presentation was 2.7 cm in size and was a Grade 3 tumor with a Ki‐67 proliferative index of 40%. Figures 2 and 3 demonstrate the initial pancreatic mass with liver metastases on imaging. She was in stage IV at this point in time based on the scoring of T2, N1, M1a using the WHO guidelines.

**FIGURE 4:**
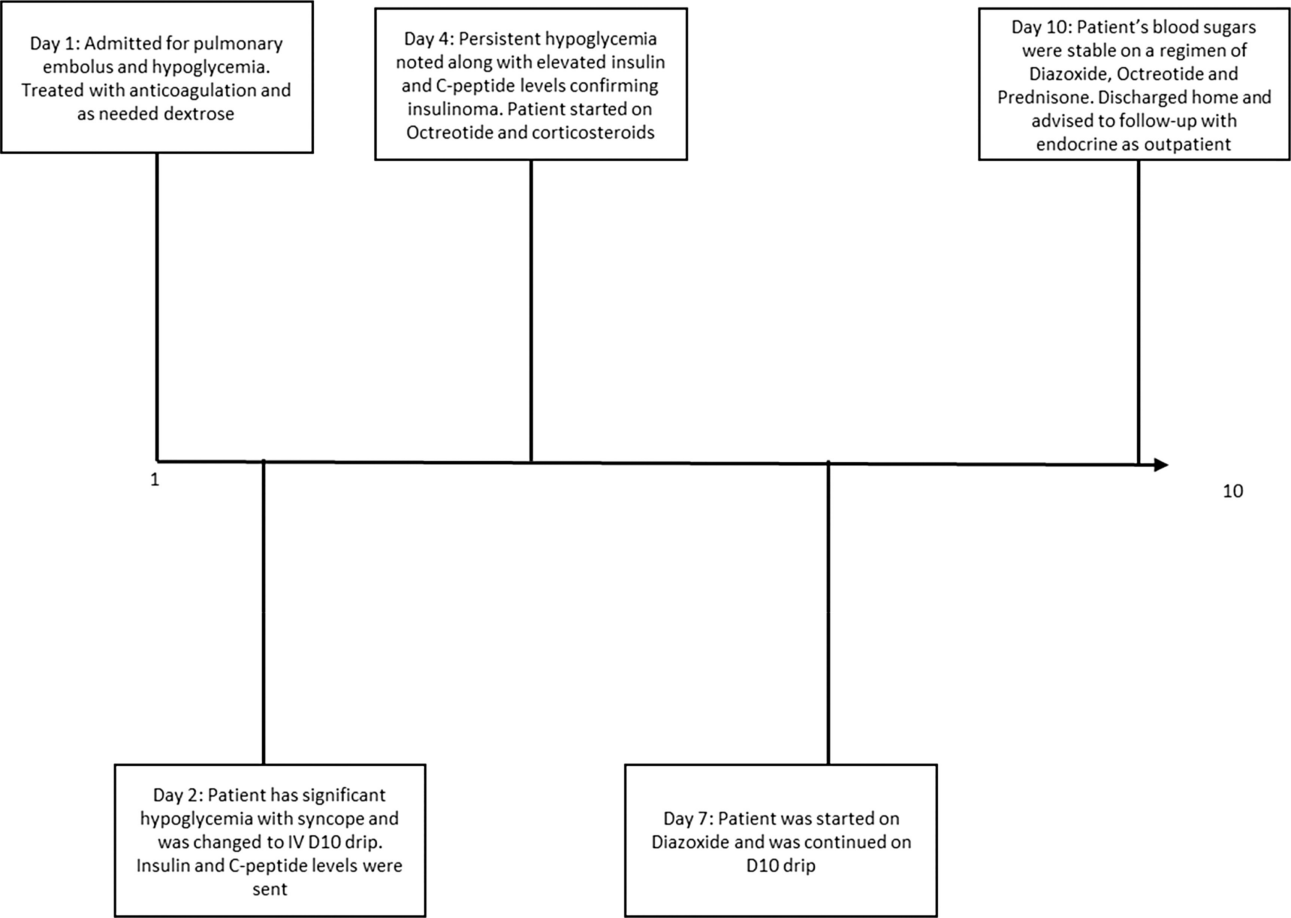
Timeline of our case presentation.

The patient continued to have persistent hypoglycemia over the next few days and was subsequently started on dextrose 10% with free water at 80 mL/h. The patient's appetite was also poor, contributing to her hypoglycemia episodes. On hospital Day 4, the patient had a syncopal episode due to a blood glucose level of 30 mg/dL. This improved with the administration of two dextrose 50 ampoules. Endocrinology workup was initiated, and the patient was started on a 72‐h fasting test. Plasma glucose was 130 mg/dL at the start of the test (normal is >70 mg/dL). The patient made it to only 2 h of fasting before becoming symptomatic. Blood glucose reached 53 mg/dL at that time, and the decision was made to discontinue the test. Blood work done at that time revealed elevated c‐peptide levels of 7.0 ng/mL (normal range is 1.1–4.4 ng/mL) and an insulin level of 136 μU/mL (normal range is 2–25 μU/mL). The patient was confirmed to have had a conversion of her high‐grade NET from nonhormone‐secreting to an insulinoma. On hospital Day 5, the patient was started on an octreotide injection of 100 mg thrice daily by the consulting endocrinologist. She was also started on dexamethasone 2 mg oral twice daily at that time. Despite these interventions, the patient continued to have frequent episodes of hypoglycemia. Her oral caloric intake had improved during this time and did not significantly contribute to hypoglycemia. At this point, the first‐line treatment for insulin‐related hypoglycemia, diazoxide, was considered by the treatment team. On hospital Day 7, the patient was started on diazoxide 50 mg po every 8 h. Her blood sugar continued to fluctuate after this treatment, and she remained on a dextrose 10% infusion. Her dexamethasone was switched to oral prednisone 40 mg daily. On this regimen, the patient's blood glucose levels did improve. She was weaned from dextrose 10% infusion with further episodes of significant hypoglycemia. She was discharged home on hospital Day 10 with a regimen of prednisone 40 mg daily and diazoxide 75 mg at night. The patient also received a one‐time chemotherapy course of folinic acid 680 mg iv, fluorouracil 4700 mg iv, and oxaliplatin 145 mg iv prior to discharge. The entire timeline of our case is shown in Figure 4.

## 3 OUTCOME/FOLLOW‐UP

The patient did establish outpatient follow‐up with oncology and endocrinology. The patient was hospitalized multiple times for recurrent hypoglycemia. After these episodes, she was maintained on octreotide continuously as an outpatient. Alternative treatment options such as everolimus and sunitinib were discussed with the patient and family, including their risks and benefits. The patient and family declined these interventions. On repeat staging imaging, the patient was noted to have a spinal lesion at T12. This was biopsied and positive for synaptophysin and chromogranin, confirming the further spread of the NET. Ki‐67 proliferative index was greater than 50%, indicating a grade 3 tumor as well.

The patient continued to do poorly overall. She declined palliative chemotherapy and was eventually placed in hospice care. The patient was managed at home with hospice services and passed away a few months later from severe hypoglycemia as a result of her insulinoma. The total survival time for our patient was 2 months from our initial evaluation in the hospital and 3 months from her initial diagnosis of a NET.

## 4 DISCUSSION

A literature review reveals a limited number of case reports that describe the conversion of a nonfunctional NET into a functional one. Multiple case series estimate the rate of conversion to be between 3.4% and 6.8%, but this date is mostly observational.^12^, ^13^ The mechanism behind this conversion remains unclear, although some studies have shown secondary conversion post‐therapy with sunitinib or traditional chemotherapy.^14^, ^15^ These studies suggest epigenetic conversion of the primary NET, which may occur because of treatment, but the mechanism is not well described. Treatment for insulinomas includes hepatic artery embolization, targeted chemoembolization, and somatostatin analogs. Hepatic artery embolization involves selective ablation of primary or secondary hepatic lesions for palliation of hypoglycemia. It has been shown to improve symptoms for at least 7 months but can even maintain this response for up to 14 months.^16^ Targeted chemoembolization of hepatic tumor sites is another similar option that alleviates hypoglycemia.^17^ The chemotherapeutic agent is selectively infused into the tumor sites leading to symptomatic relief. These treatments do not seem to last as long, however, and need to be repeated often to maintain efficacy over multiple months or years.^18^ Somatostatin analogs such as octreotide and lanreotide have been shown to decrease symptoms and slow progression^19^ but are generally only used in the short term. Among the selective newer treatment options for malignant insulinoma are everolimus and sunitinib. Everolimus inhibits the MTOR pathway that is a part of insulin‐related gluconeogenesis and is effective as an adjunctive therapy in patients who are not a candidate for surgical treatment.^20^ Sunitinib is a tyrosine kinase inhibitor that directly inhibits tumor growth, thereby reducing insulin production, but it can sometimes cause paradoxical hypoglycemia on its own.^14^, ^21^ Malignant insulinomas carry a good prognosis with a 5‐year survival rate of 62% and a medium survival time of 40 months in patients with low‐grade tumors doing much better.^22^ These patients also benefit from new therapies and often reach remission. However, patients with high‐grade tumors and/or distant metastatic disease do significantly worse with a median survival time less than 24 months.^6^, ^23^

Our patient was already diagnosed with metastatic pancreatic NET in the outpatient setting prior to presentation. She had known metastases to the liver and lungs when she was admitted for management of acute pulmonary embolism. This tumor had tested positive for NET tumor markers of synaptophysin and chromogranin on initial evaluation. It is not common for functional testing to occur for tumors unless the patient demonstrates any clinical signs of a functional tumor, which is what this case report describes. While hospitalized, the patient developed symptomatic hypoglycemia with decreased blood glucose levels that improved with glucose administration. These three clinical findings, also known as Whipple's triad,^4^ strongly raised the suspicion for conversion of the primary pancreatic NET into an insulinoma. Subsequent measurements of fasting insulin and c‐peptide levels confirmed this diagnosis. Interestingly, our patient did not receive any treatment prior to conversion and had a much shorter time of conversion than is described in the literature, 3 months versus a median of 15 months in other reports.^12^ Due to her metastatic disease prior to conversion, treatment options remained limited. Hepatic artery embolization and targeted chemoembolization were not offered due to the presence of lung and spinal metastases. She was hospitalized numerous times for insulin‐related complications and was not a candidate for any other aggressive therapy. As a result of limited therapy, our patient also had a quick decline, surviving only 3 months in total.

Our case describes one of the few instances of conversion from a nonfunctional NET into an insulinoma without any prior treatment and over a much shorter time frame. It also describes an aggressive clinical course in these patients due to uncontrolled symptoms related to hypoglycemia and a paucity of treatment options therein. Traditional insulinoma treatments, including surgical resection, could have been utilized, but metastatic disease made this impossible. Somatostatin analogs such as octreotide were utilized without significant sustained benefit. Newer drugs such as everolimus and sunitinib could have been an option for our patient but were not an option as the patient declined these treatments. There may have been some hesitation due to the novelty of these treatments and a lack of strong evidence in their favor.

## 5 CONCLUSION

Our case report involves the conversion of a metastatic nonfunctional NET into a functional malignant insulinoma. The key clinical points from this case report are as follows: (1) when a nonfunctional NET converts into a functional one, the prognosis worsens significantly; (2) alternative treatments beyond conventional chemotherapy are required for patients with aggressive insulinomas; and (3) more research is required to evaluate treatments such as everolimus and sunitinib for their use in cases of malignant insulinomas.

## AUTHOR CONTRIBUTIONS

**Venkata S. Buddhavarapu:** Data curation; formal analysis; investigation; methodology; writing – original draft. **Gagandeep Dhillon:** Formal analysis; resources; writing – review and editing. **Harpreet Singh Grewal:** Formal analysis; writing – review and editing. **Brian Soles:** Data curation; formal analysis; investigation. **Luke Halbur:** Conceptualization; data curation; formal analysis. **Salim Surani:** Project administration; validation; writing – review and editing. **Rahul Kashyap:** Formal analysis; investigation; project administration; writing – review and editing.

## FUNDING INFORMATION

Publishing and Texas A&M University have an agreement that authors would not be responsible for the APC.

## CONFLICT OF INTEREST STATEMENT

None of the coauthors have reported any conflict of interest.

## CONSENT

Written informed consent was obtained from the patient to publish this report in accordance with the journal's patient consent policy.

## Data Availability

Not applicable to protect patient privacy for this case report manuscript.
